# A plasma proteomic signature of the actin-coagulation axis accurately predicts progression to active tuberculosis

**DOI:** 10.3389/fmicb.2025.1746190

**Published:** 2026-01-26

**Authors:** Peng Lu, Weiwei Gao, Xiaoyan Ding, Jingjing Pan, Hui Ding, Qiao Liu, Limei Zhu, Xiang Huo

**Affiliations:** 1 Department of Chronic Communicable Disease, Jiangsu Provincial Center for Disease Control and Prevention, Nanjing, China; 2 Key Laboratory of Public Health Safety and Emergency Prevention and Control Technology of Higher Education Institutions in Jiangsu Province, Department of Epidemiology, School of Public Health, Nanjing Medical University, Nanjing, China; 3 Department of Tuberculosis, The Second Hospital of Nanjing, Nanjing University of Chinese Medicine, Nanjing, China

**Keywords:** actin cytoskeleton, coagulation, prognostic biomarker, proteomics, tuberculosis

## Abstract

**Background:**

Predicting progression to active tuberculosis (TB) is a critical unmet need, as current immunological tests only detect infection but cannot discriminate those who will develop active disease. A reliable prognostic biomarker could enable targeted preventive therapy and transform TB control strategies.

**Methods:**

We performed deep plasma proteomics using data-independent acquisition mass spectrometry on a prospective cohort of 60 TB-exposed students in Jiangsu Province, China, comprising 40 individuals with *Mycobacterium tuberculosis* infection and 20 uninfected controls.

**Results:**

Over 2 years of follow-up, 21 of the 40 infected participants progressed to active disease, and a LASSO-Cox model with internal cross-validation for tuning the regularization parameter (*λ*) was used to develop a prognostic signature from baseline plasma samples. A 33-protein signature predicted progression to active TB with high discriminatory performance in this cohort (AUC = 0.992, 95% CI 0.977–1.0). This signature revealed a distinct pre-symptomatic state in progressors, defined by a pro-thrombotic shift in the coagulation cascade and profound disruption of actin cytoskeleton dynamics. The proteomic alterations were detectable up to 2 years before clinical diagnosis, providing a potential window for intervention. Functional network analysis identified key hub proteins including ACTR3, ACTN1, and MYH9 (actin remodeling) and F2 (coagulation).

**Conclusion:**

We identified a plasma protein signature that accurately predicts progression from latent to active tuberculosis, linking disease onset to dysregulation of the actin cytoskeleton and coagulation. This biomarker provides a basis for precision preventive therapy and identifies novel host-directed therapeutic targets.

## Introduction

Tuberculosis (TB), caused by *Mycobacterium tuberculosis* (*M.tb*), remains one of the leading infectious causes of mortality worldwide, with an estimated 10.8 million new cases reported in 2023. A major obstacle to TB elimination is the vast pool of individuals harboring *M.tb* infection-roughly a quarter of the global population (World Health Organization, 2024). Although most will never develop active disease, about 5–10% progress during their lifetime, sustaining ongoing transmission (Riza et al., 2014). Preventive treatment of infected individuals is central to TB control; however, current approaches are undermined by the inability to identify with precision those most likely to develop disease.

The Tuberculin Skin Test (TST) and Interferon-*γ* Release Assays (IGRAs), the main tools for detecting *M.tb* infection, measure adaptive immune responses to mycobacterial antigens but are limited in scope. Neither test discriminates active TB from latent infection, nor can they determine whether an infection will remain contained or advance to clinical disease (Togun et al., 2020). Their poor positive predictive value for progression leads to widespread overtreatment, exposing individuals to drug toxicities, including hepatotoxicity, and straining healthcare systems (Shah and Dorman, 2021). The development of a reliable, non–sputum-based biomarker that can prospectively identify individuals at imminent risk of TB is therefore a critical global priority, with the potential to enable targeted preventive therapy and reshape control strategies (Huerga et al., 2023).

The immunological response to *M.tb* infection is dynamic, with molecular perturbations emerging well before clinical disease becomes apparent. Capturing these early changes offers a unique opportunity for risk stratification. While transcriptional signatures have provided proof of concept (Zak et al., 2016), proteomic profiling may more directly capture functional immune states, as proteins represent the active effectors of biological pathways. Plasma, in particular, is an attractive substrate for biomarker discovery due to its accessibility and its ability to reflect systemic pathophysiology (Palstrøm et al., 2022). Recent advances in high-resolution, Data-independent acquisition (DIA) mass spectrometry now enable reproducible, quantitative interrogation of the plasma proteome at depth, creating new opportunities for robust biomarker discovery (Muneer et al., 2025).

In this prospective cohort study of student close contacts, a population with well-defined elevated risk, we applied DIA mass spectrometry-based proteomics with two aims. First, to define a plasma protein signature that reliably distinguishes active TB from *M.tb* infection and uninfected controls. Second, and most critically, to identify and evaluate prognostic signature predictive of progression from latent infection to active disease. Our overarching goal is to lay the molecular foundation for a blood-based diagnostic capable of guiding precision preventive therapy in individuals with *M.tb* infection.

## Methods

### Study design and participants

This study was nested within a prospective cohort established in Jiangsu Province, China, between 2020 and 2021. We selected a cohort of students identified during a school-based TB contact investigation, and the age distribution reflects the demographic structure of the exposed student population. At baseline, all participants underwent screening with a TST and chest X-ray. An IGRA (QuantiFERON-TB Gold-in Tube, QIAGEN) was used to definitively establish *M.tb* infection status. Residual plasma from IGRA testing was cryopreserved at −80 °C for subsequent biomarker analysis. Plasma samples used for proteomic analysis were collected at baseline only; follow-up visits were used for clinical monitoring and outcome ascertainment.

For this proteomic study, we selected a cohort of 60 individuals and defined three mutually exclusive groups using a single categorical variable (group = 0/1/2): G0 (IGRA-negative controls, *N* = 20), G1 (IGRA-positive non-progressors, *N* = 19), and G2 (IGRA-positive progressors, *N* = 21). These group definitions and sample sizes are used consistently throughout the manuscript.

### Follow-up and case ascertainment

All participants were prospectively monitored for a 2 year period to identify incident cases of active TB. Follow-up involved biannual symptom screening by school and local public health staff. To ensure comprehensive case ascertainment, this prospective data was cross-referenced with the provincial Tuberculosis Management Information System for 2020–2022 using unique personal identifiers. A diagnosis of active TB was confirmed according to national diagnostic criteria.

### Plasma proteomics

#### Sample preparation

High-abundance proteins (albumin, IgG) were depleted from plasma samples using an immunoaffinity column (Thermo Fisher Scientific). The depleted plasma was then digested into peptides using a filter-aided sample preparation (FASP) protocol with sequencing-grade trypsin. Resulting peptides were desalted via C18 solid-phase extraction.

#### LC-MS/MS analysis

A project-specific spectral library was first generated by analyzing 10 high-pH reversed-phase fractions of a pooled sample in Data-dependent acquisition (DDA) mode. For individual sample analysis, peptides were separated on a nano-LC system (EASY-nLC 1200) and analyzed on an Orbitrap Exploris 480 mass spectrometer (Thermo Fisher Scientific) using a DIA strategy with 45 variable isolation windows.

#### Data processing

Raw DIA files were processed against the spectral library using Spectronaut (v.18.0, Biognosys AG). Protein identification was accepted at a False discovery rate (FDR) of <1% at both the precursor and protein levels. Protein quantification data were then exported for statistical analysis.

### Quality control

System stability and data quality were monitored throughout the acquisition sequence. Technical quality control metrics, including retention time stability across runs (inter-run iRT delta < 0.5 min) and consistent chromatographic peak shape (mean full width at half maximum [FWHM] ≈ 0.25 min), were assessed to ensure high data reliability and reproducibility.

### Statistical analysis

All statistical analyses were conducted in R (version 4.3.1), and a two-sided *p*-value < 0.05 was considered statistically significant.

### Proteomic data processing and quality control

Raw protein intensities were filtered to retain proteins with at least 50% quantification across all samples. Any remaining missing values were imputed using half the minimum observed value for the respective protein. The imputed data were then sequentially log₂-transformed and *Z*-score normalization was used for visualization (e.g., heatmaps/PCA) rather than for differential expression testing. The high reproducibility of the dataset was confirmed by intra-group Pearson correlation analysis.

### Differential expression and functional pathway analysis

To delineate proteomic changes across the tuberculosis spectrum, linear models were fitted for each protein using the limma R package, which employs an empirical Bayes method to moderate standard errors. Proteins were deemed significantly dysregulated if they met a FDR adjusted *p*-value < 0.05 and an absolute fold-change > 1.2. Gene Ontology (GO) enrichment analysis was performed on lists of significant proteins using clusterProfiler, with all quantified proteins serving as the background set. Protein–protein interaction (PPI) networks were constructed from key prognostic proteins using the STRING database (v11.5), retaining only high-confidence interactions (score > 0.7). Network topology and hub proteins were identified using the igraph package.

### Development and evaluate of a prognostic signature

A prognostic signature to predict progression to active tuberculosis was developed using baseline data from the 40 participants with latent *M.tb* infection. We employed a LASSO-penalized Cox proportional hazards regression model. The regularization parameter (*λ*) was selected using internal cross-validation as implemented in cv.glmnet (glmnet package). Model performance was evaluated within this discovery cohort using the Area under the receiver operating characteristic curve (AUC) with 95% confidence intervals. Given the absence of an independent validation cohort, the reported performance metrics should be interpreted as internal estimates.

### Performance assessment of the prognostic signature

The discriminatory performance of the model-derived risk scores was assessed by the AUC with 95% confidence intervals. These performance estimates reflect internal evaluation within this discovery cohort. For survival analysis, participants were stratified into high- and low-risk groups based on the median risk score, and differences in tuberculosis-free survival were evaluated using the log-rank test. The clinical utility of the model was quantified by Decision curve analysis (DCA), which estimates the net benefit across a range of clinically relevant risk thresholds. Finally, principal component analysis (PCA) was used to visualize the unsupervised separation of progressors and non-progressors based on the signature proteins. A multi-class random forest classifier was also developed to assess the diagnostic potential of the proteomic data.

### Ethics approval and consent to participate

This study was reviewed and approved by Department of Science and Technology, Nanjing Medical University. All eligible participants signed written informed consent (Approval Number: 2019(225)).

## Results

### Cohort characteristics and global proteomic landscape of tuberculosis progression

The study was based on a cohort of 60 participants, including G0 (*N* = 20), G1 (*N* = 19), and G2 (*N* = 21) participants defined by baseline IGRA status and 2 year outcomes. The baseline demographic and clinical characteristics of the cohort, stratified by final outcome, are presented in Table 1 and Supplementary Figure 1. The groups were well-matched for age and sex, providing a robust basis for subsequent proteomic comparisons.

**Table 1:** Demographic and clinical characteristics of participants by incident TB disease status (*n* = 60).

	G0	G1	G2
*N*	20	19	21
Age [mean (SD)]	26.65 ± 8.37	20.74 ± 0.73	20.86 ± 3.14
Sex (%)			
Female	15 (75.0%)	13 (78.9%)	7 (66.7%)
Male	5 (25.0%)	4 (21.1%)	7 (33.3%)
ACTR3 [mean (SD)]	64956.6 ± 44870.5	64956.6 ± 44870.5	64956.6 ± 44870.5
HSP90AA1 [mean (SD)]	67645.5 ± 42095.1	67645.5 ± 42095.1	67645.5 ± 42095.1
HSPA8 [mean (SD)]	391992.8 ± 230727.7	391992.8 ± 230727.7	391992.8 ± 230727.7
ARPC2 [mean (SD)]	103353.2 ± 67145.3	103353.2 ± 67145.3	103353.2 ± 67145.3
F2 [mean (SD)]	108158444.8 ± 8939020.5	108158444.8 ± 8939020.5	108158444.8 ± 8939020.5
MYL6 [mean (SD)]	398360.0 ± 328116.4	398360.0 ± 328116.4	398360.0 ± 328116.4
MYH9 [mean (SD)]	118828.3 ± 118220.7	118828.3 ± 118220.7	118828.3 ± 118220.7
CAPZB [mean (SD)]	60774.3 ± 35355.6	60774.3 ± 35355.6	60774.3 ± 35355.6
SEPTIN7 [mean (SD)]	30273.0 ± 24646.2	30273.0 ± 24646.2	30273.0 ± 24646.2
ARPC5 [mean (SD)]	162407.1 ± 95365.2	162407.1 ± 95365.2	162407.1 ± 95365.2

G0: IGRA- controls, G1: IGRA+ non-tuberculosis, G2: IGRA+ active tuberculosis.

To delineate the plasma proteomic signatures of tuberculosis progression, we performed DIA mass spectrometry on all baseline plasma samples. Quality control assessment confirmed the high quality and reproducibility of the dataset, with intra-group Pearson correlation coefficients consistently exceeding 0.95. Sequential normalization effectively corrected the right-skewed data distribution, rendering it suitable for statistical analysis (Supplementary Figure 2).

**Figure 1:**
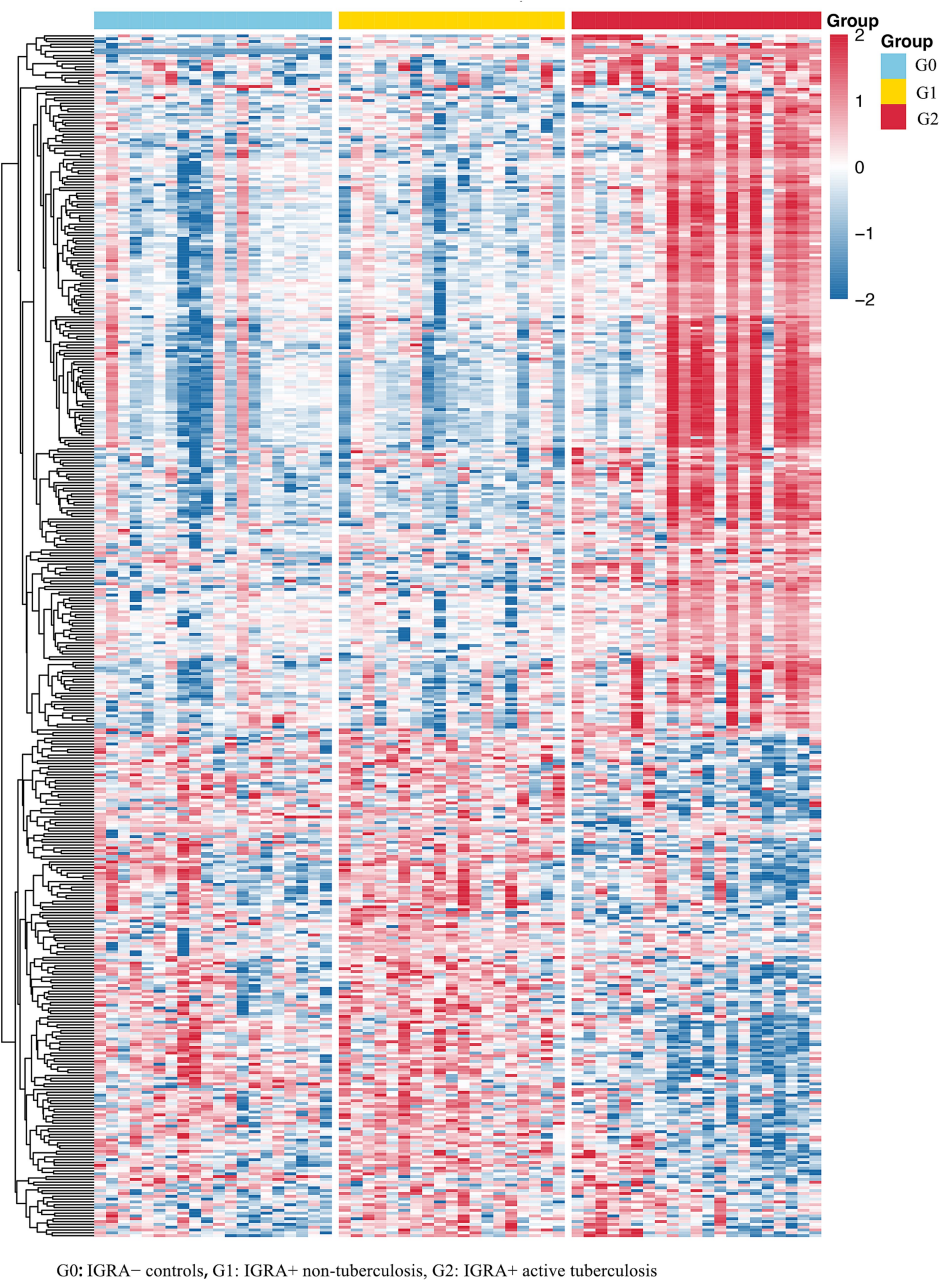
Hierarchical clustering reveals progressively distinct plasma proteomic signatures across the tuberculosis disease spectrum.

Using two pre-specified pairwise comparisons reflecting infection-associated and progression-associated transitions (G1 vs. G0 and G2 vs. G1) and defining differentially expressed proteins (DEPs) as *p* < 0.05 and FC > 1.2 (|log2FC| > 0.263), we identified 428 unique DEPs across these transitions. Hierarchical clustering based on these 428 DEPs revealed a stepwise separation of G0, G1, and G2 proteomic profiles, consistent with progressive remodeling of the plasma proteome from uninfected status to latent infection and onward to active TB (Figure 1).

**Figure 2:**
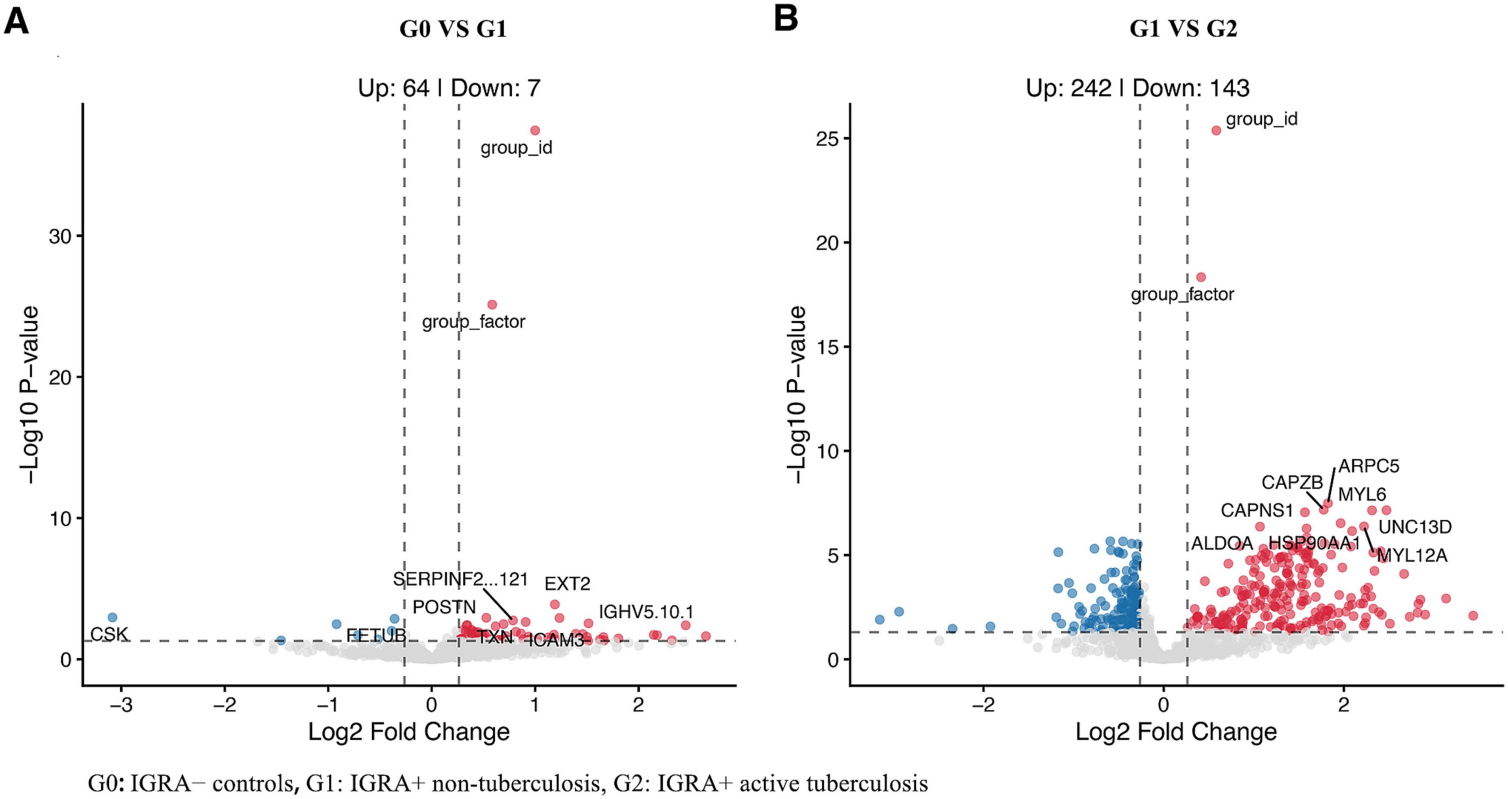
Volcano plots illustrating the escalating magnitude of plasma proteomic dysregulation across the tuberculosis progression continuum. **(A)** Volcano plot showing differentially expressed proteins between healthy controls and latent infection (G0 vs G1). **(B)** Volcano plot showing differentially expressed proteins between latent infection and active tuberculosis (G1 vs G2).

Differential expression analysis further demonstrated that the transition from G0 to G1 was characterized by a relatively contained host response, with 71 DEPs (64 up-regulated and 7 down-regulated) (Figure 2A). In contrast, progress from G1 to G2 was associated with substantially broader proteomic perturbation, with 385 DEPs (242 up-regulated and 143 down-regulated) (Figure 2B), including prominent changes in cytoskeletal and hemostatic-related proteins.

**Figure 3:**
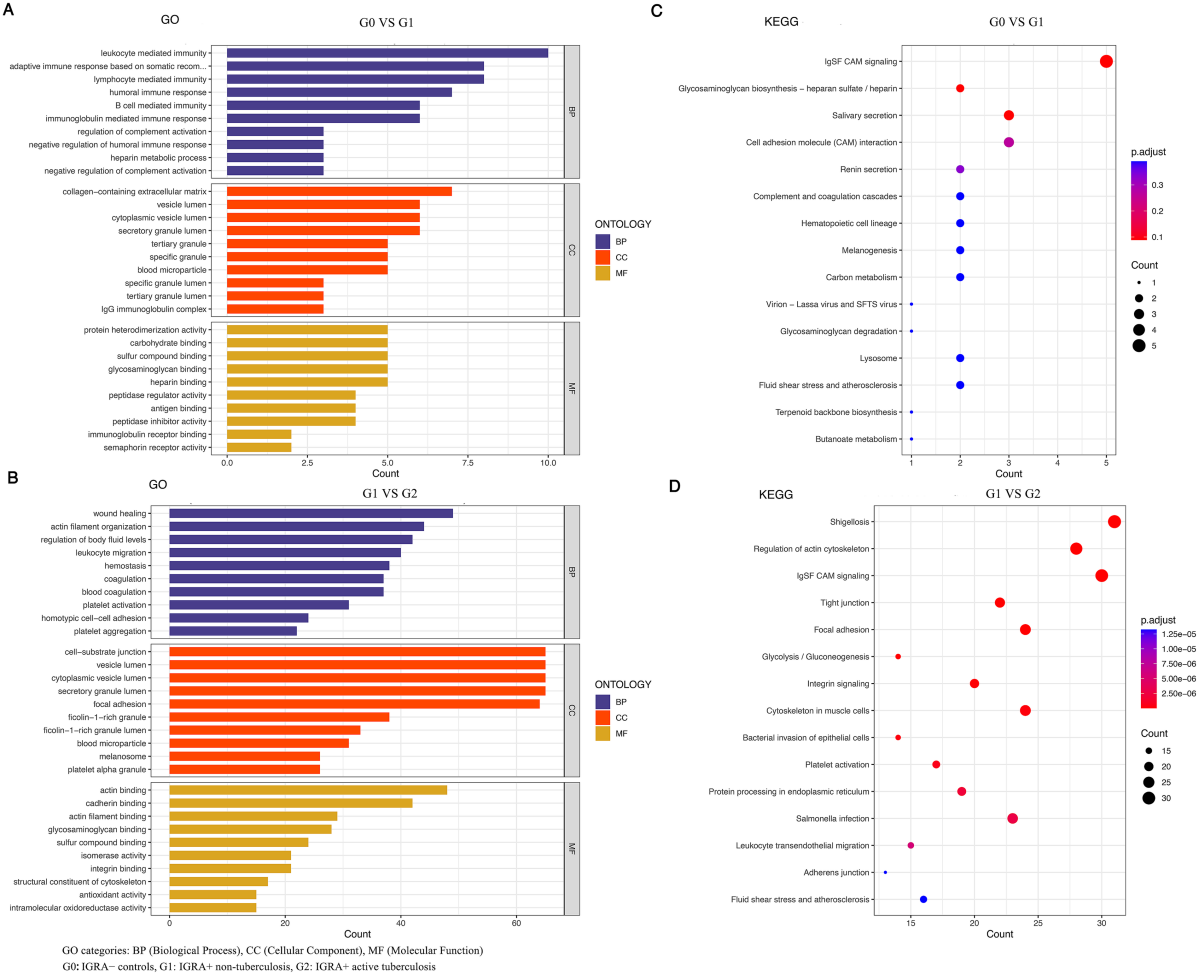
Distinct functional pathways underpin the transition from latent infection to active tuberculosis. **(A)** Gene Ontology (GO) enrichment analysis of differentially expressed proteins for G0 vs G1. **(B)** Gene Ontology (GO) enrichment analysis of differentially expressed proteins for G1 vs G2. **(C)** KEGG pathway enrichment analysis for the G0 vs G1 comparison. **(D)** KEGG pathway enrichment analysis for the G1 vs G2 comparison.

Functional enrichment analysis highlighted distinct biological processes underlying infection versus progression (Figure 3). For G1 vs. G0, enriched terms were dominated by immune-related processes, including leukocyte-mediated and humoral immune responses and regulation of complement activation (Figure 3A), with KEGG pathways including complement and coagulation cascades and related host-response pathways (Figure 3C). For G2 vs. G1, enrichment shifted toward pathways implicated in tissue remodeling and hemostasis, including actin cytoskeleton remodeling, platelet activation, and cell adhesion programs (Figures 3B,D), supporting an actin-coagulation axis as a key feature associated with progression to active TB.

To explore the diagnostic potential of these proteomic profiles, we trained a multi-class random forest classifier to distinguish among G0, G1, and G2. The model showed good cross-validation performance, achieving a mean AUC of 0.875, with particularly strong discrimination for the active TB/progressor group (class-specific AUC of 0.969) (Supplementary Figure 3).

### A baseline proteomic signature accurately predicts progression to active TB

**Figure 4:**
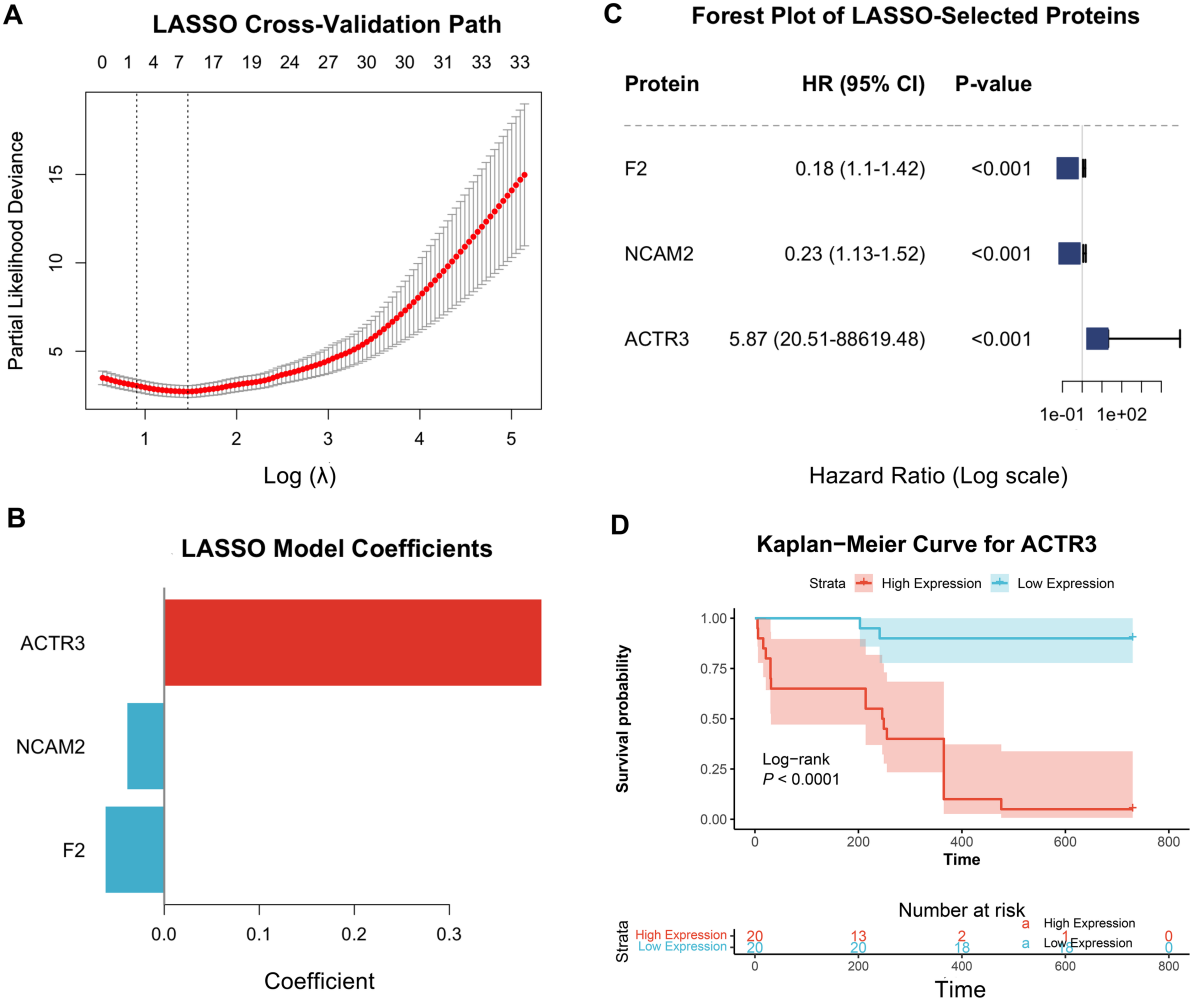
Development and characterization of a LASSO-Cox-derived proteomic signature for predicting progression to active tuberculosis. **(A)** Selection of the optimal tuning parameter (λ) using LASSO cross-validation. **(B)** LASSO coefficient profiles of the three selected prognostic proteins (ACTR3, NCAM2, F2). **(C)** Forest plot displaying the Hazard Ratios (HR) and p-values for the selected protein signature. **(D)** Kaplan-Meier survival curve stratified by high versus low expression of ACTR3.

Building on these findings, we developed a prognostic model to predict TB progression using the baseline proteomic data from the 40 individuals with latent *M.tb* infection. A LASSO-Cox regression model with 10-fold cross-validation identified an optimal signature comprising 33 proteins (Figures 4A,B). This signature included powerful risk amplifiers like ACTR3 (HR = 5.87) and protective factors such as F2 (HR = 0.18) (Figure 4C). The prognostic value of key markers was evident in survival analysis; for instance, high baseline expression of ACTR3 was strongly associated with a significantly shorter time to tuberculosis diagnosis (Log-rank *p* < 0.0001; Figure 4D).

The high discriminatory power of this 33-protein signature was visualized by principal component analysis. The first two principal components explained 74.7 and 16.7% of the variance, respectively, and achieved a clear and robust separation of progressors from non-progressors based solely on their baseline proteomic profiles (Supplementary Figure 4). Furthermore, DCA confirmed the signature’s substantial clinical utility. The model provided a greater net benefit for clinical decision-making across a wide range of risk thresholds compared to the default strategies of treating all or no individuals (Supplementary Figure 5).

### The prognostic signature reveals a core pathological network

**Figure 5:**
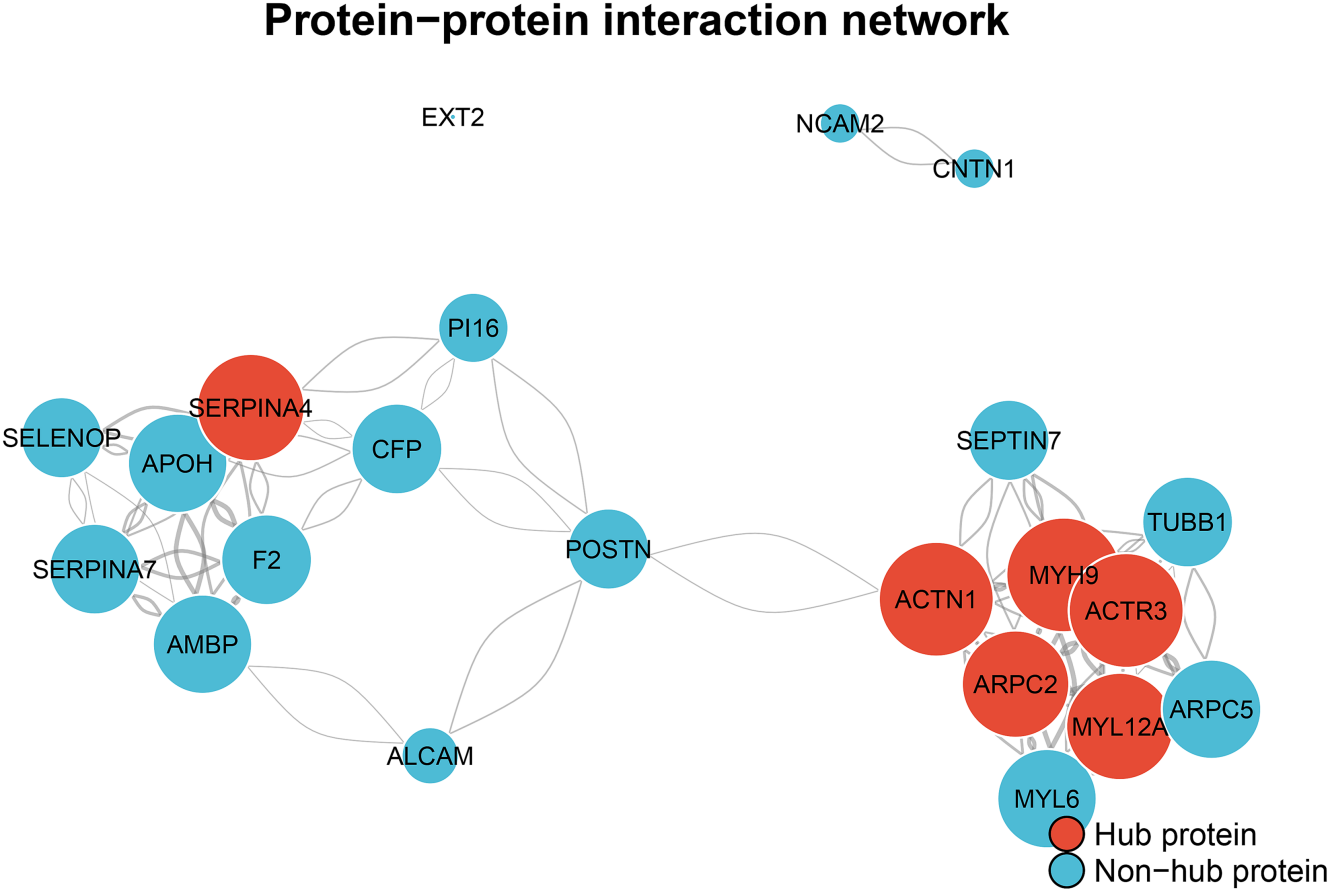
The prognostic proteomic signature forms interconnected pathological axes of actin cytoskeleton remodeling and coagulopathy.

To elucidate the mechanistic basis of our prognostic signature, we constructed a PPI\ network using 23 of the most inter-connected LASSO-selected proteins. The analysis revealed that these predictive markers form a highly integrated biological system comprising 22 nodes and 108 edges, organized around two central pathological axes: actin cytoskeleton remodeling and coagulopathy (Figure 5). The largest module was centered on cytoskeletal dynamics, featuring highly connected hub proteins such as ACTN1 (degree = 16), MYH9 (degree = 16), and ACTR3 (degree = 16). This hub was linked to a coagulation-inflammation module involving F2 (thrombin) and SERPINA4. This network provides a compelling mechanistic model, suggesting that the signature’s predictive power derives from its ability to detect the early activation of these interconnected pathways.

## Discussion

In this prospective study of a high-risk cohort, we harnessed advanced quantitative proteomics to dissect the molecular underpinnings of TB progression. Our analysis has uncovered a plasma protein signature that not only discriminates active TB from *M.tb* infection and uninfected controls and forecasts progression with high apparent discrimination in this cohort. These insights help address a longstanding gap in global TB control, laying the foundation for a blood-based assay to inform targeted preventive therapies and support strategies for averting active disease.

A cornerstone of our findings is the marked reconfiguration of the plasma proteome as *M.tb* infection tips into active TB, spotlighting profound derangements in actin cytoskeleton regulation and coagulation cascades as pivotal engines of this shift. Biologically, this resonates deeply: macrophages, the frontline arena of TB pathogenesis, depend on orchestrated actin remodeling for phagocytosis, phagosome maturation, autophagy, and granuloma fortification. *M.tb* may interfere with these mechanisms to dodge immune surveillance, and our proteome data expose systemic imbalances in actin modulators like ARPC5 and ACTN1, signaling an unraveling of cellular restraint (Naish et al., 2023; Palacios et al., 2021). Such alterations likely echo faltering macrophage efficacy or eroding granuloma barriers-the very fulcrum where bacterial containment collapses, paving the way for dissemination-echoing observations from cellular models where *M.tb* effectors disrupt host actin networks to favor intracellular survival (Dutta et al., 2022; Hill and Welch, 2022).

Running parallel is the striking coagulation fingerprint we delineated, which dovetails with the entrenched phenomenon of “immunothrombosis” in active TB-a feedback loop wherein inflammation begets clotting, and clotting fans inflammation (Chen et al., 2022; Caccamo and Dieli, 2016). Hitherto regarded as a hallmark of late-stage illness (Jorgensen et al., 2020), our results recast this hypercoagulable milieu as an early harbinger, antedating overt symptoms. This coagulopathy emerges as an active accomplice in progression, perhaps cocooning bacilli in fibrin scaffolds or inciting microvascular occlusion and tissue injury-contrasting with prior views that pegged it chiefly to hypoxic lung damage in cavitary disease, and aligning instead with emerging evidence of its role in granuloma consolidation and immune containment (Donohue et al., 2025). Together, these findings suggest that coagulation-related perturbations may occur earlier in TB pathogenesis than previously appreciated and warrant further mechanistic investigation (Kager et al., 2015).

The quest for a reliable prognostic biomarker for TB progression has been a global research priority for over a decade. Seminal work on blood RNA signatures demonstrated that subclinical disease activity is detectable before diagnosis (Paton et al., 2023), but their performance varies across populations, and their complexity hampers clinical adoption (Kreitmann et al., 2024). Meanwhile, lone sentinels like C-reactive protein (CRP), IP-10, or IL-6-linked cytokines have surfaced as progression harbingers (Zimmer et al., 2022; LaVergne et al., 2020; Zhang et al., 2024), but their modest specificity often demands multiplexing, as standalone metrics falter in heterogeneous settings-unlike our multi-analyte panel, which weaves pathway crosstalk for sharper resolution. Earlier proteomic forays in TB were mostly snapshots, unmasking diagnostic flags but skirting progression dynamics. Our forward-looking cohort design bridges that chasm. Moreover, the model achieved an AUC of 0.992 as an internal performance estimate within this cohort. However, given the modest sample size, the homogeneous young adult population, and the lack of an independent validation cohort, these performance estimates should be interpreted as preliminary and require confirmation in larger, independent cohorts. DCA further cements clinical promise, quantifying net gains over blanket treatment or inaction across realistic risk strata. This propels our work from benchside revelation to frontline feasibility, contrasting with RNA tools’ logistical barriers in resource-scarce locales (Drain et al., 2018).

Transcending diagnostics, our signature’s proteins beckon as footholds for host-directed therapies. The actin-coagulation nexus hints at bolstering macrophage resilience or blunting TB-linked thrombophilia to stall advance-take ARP2/3 targeting (via ARPC2/ARPC5) to mend cytoskeletal breaches, or repurposed statins/aspirin for dual anti-inflammatory/antithrombotic punch, building on their proven dampening of endothelial activation in inflammatory states (Caccamo and Dieli, 2016; Ghosh et al., 2013; Mitroi et al., 2025). Such tactics dovetail with evolving infectious disease doctrines, blending antimicrobials with host tweaks to rein in runaway inflammation (Wallis and Hafner, 2015; de Isla et al., 2005).

Our study’s strengths include its prospective design in a well-defined cohort of recently exposed individuals, ideal for studying progression, and the use of cutting-edge DIA mass spectrometry for high-quality, reproducible data.

Our study has several limitations. First, the modest sample size necessitates corroboration in larger, multi-site prospective trials to ensure the robustness and reproducibility of the identified signature. Second, we did not include an independent external validation cohort. This constraint reflects the epidemiological context of our study setting in Jiangsu Province, a relatively low-incidence region for tuberculosis, where accruing a sufficient number of additional progressors for a second prospective cohort within a limited timeframe is challenging. Therefore, independent validation in larger cohorts—particularly in higher-burden settings and populations with broader demographic and clinical diversity—is essential before clinical translation. Third, our study population consisted primarily of young adults identified during a school-based contact investigation, which explains the narrow age distribution. While this demographic likely reduces confounding from age-related comorbidities, it limits generalizability to older populations and to individuals with complex clinical backgrounds (e.g., HIV co-infection, diabetes, or undernutrition). Furthermore, due to the nature of an emergency public health investigation, detailed baseline health metrics such as BMI and systematic assessment of comorbidities were not mandatorily collected and thus could not be incorporated into the present analyses. Finally, while our network analysis highlights the actin–coagulation axis as a key feature of progression, the current study is observational and establishes association rather than causality; mechanistic studies *in vitro* and *in vivo* will be required to clarify the functional roles of these pathways and to evaluate whether they represent actionable targets for host-directed intervention. Accordingly, the reported model performance should be interpreted as hypothesis-generating and as an internal estimate within this discovery cohort.

In conclusion, we identify a plasma proteomic signature associated with progression from *M.tb* infection to active TB in this discovery cohort. The enrichment of pathways linking actin cytoskeleton regulation and coagulation supports an actin-coagulation axis as a key feature of progression and highlights candidate host pathways for intervention. Future work should prioritize validation in larger and more diverse independent cohorts and development of a scalable assay suitable for risk stratification in preventive programs.

## Data Availability

The mass spectrometry proteomics data have been deposited to the ProteomeXchange Consortium (https://proteomecentral.proteomexchange.org) via the iProX partner repository with the dataset identifier PXD072897.

## Supplementary material

SUPPLEMENTARY FIGURE 1 Group-wise distribution of selected plasma proteins across the TB spectrum.

SUPPLEMENTARY FIGURE 2 Preprocessing and normalization pipeline for the quantitative plasma proteomic data. **(A)** The right-skewed distribution of raw protein intensities. **(B)** The distribution after log₂ transformation. **(C)** The final approximately normal distribution after z-score normalization.

SUPPLEMENTARY FIGURE 3 Cross-validation performance of a random forest classifier for distinguishing among active tuberculosis, latent infection, and healthy states. **(A)** Confusion matrix showing classification accuracy for each class. **(B)** Top 10 most important protein features ranked by their contribution to the model. **(C)** Receiver operating characteristic (ROC) curves for each class, with corresponding area under the curve (AUC) values. **(D)** Boxplots of predicted probabilities for each true group.

SUPPLEMENTARY FIGURE 4 Principal component analysis based on the prognostic proteomic signature demonstrates robust separation of individuals with latent infection and active tuberculosis.

SUPPLEMENTARY FIGURE 5 Decision curve analysis confirms the clinical utility of the prognostic proteomic signature for guiding intervention decisions.
