# Supplementary figures and images for: A plasma proteomic signature of the actin-coagulation axis accurately predicts progression to active tuberculosis
Source: Front Microbiol. 2026 Jan 26;16:1746190. doi: 10.3389/fmicb.2025.1746190 (PMC12883649; doi:10.3389/fmicb.2025.1746190)

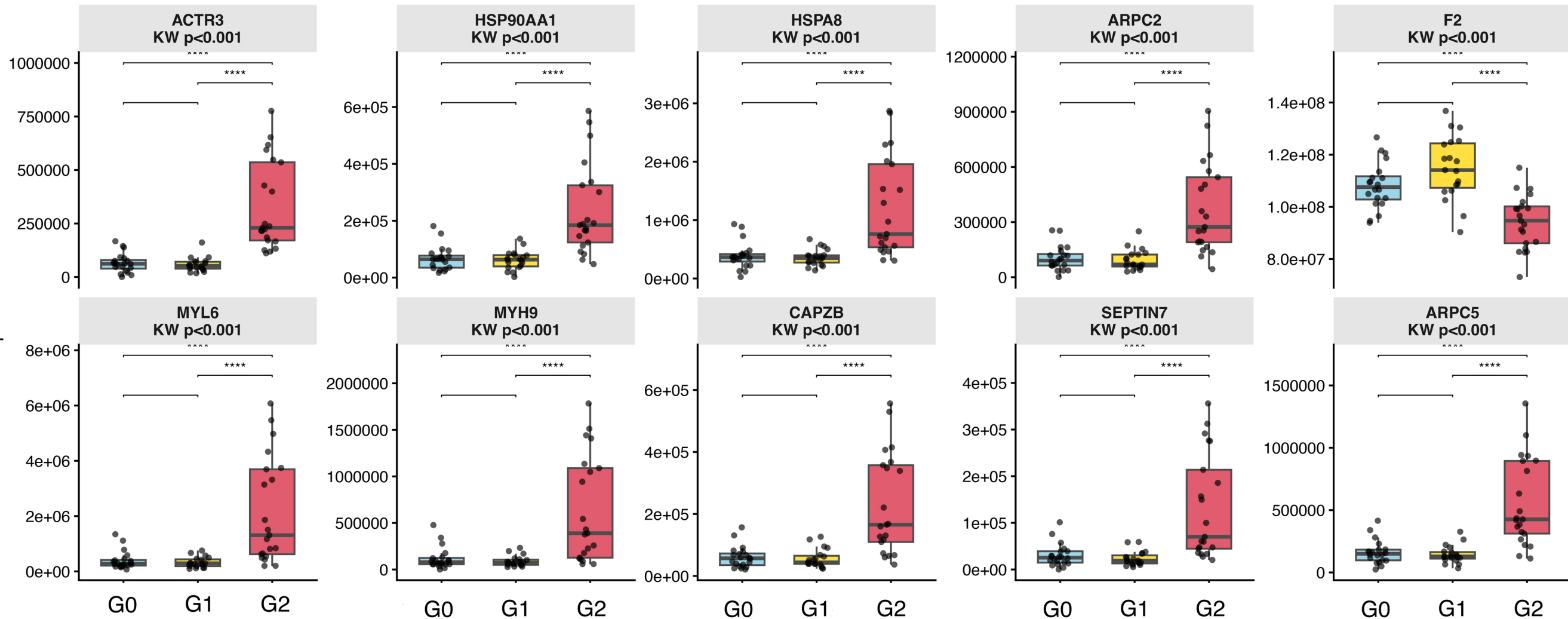

G0: IGRA- controls, G1: IGRA+ non-tuberculosis, G2: IGRA+ active tuberculosis

Supplement: SUPPLEMENTARY FIGURE 1 — Group-wise distribution of selected plasma proteins across the TB spectrum. [file Data_Sheet_1.pdf]

# PCA of Selected Proteins

Group

● *M.tb* infection

● Active tuberculosis

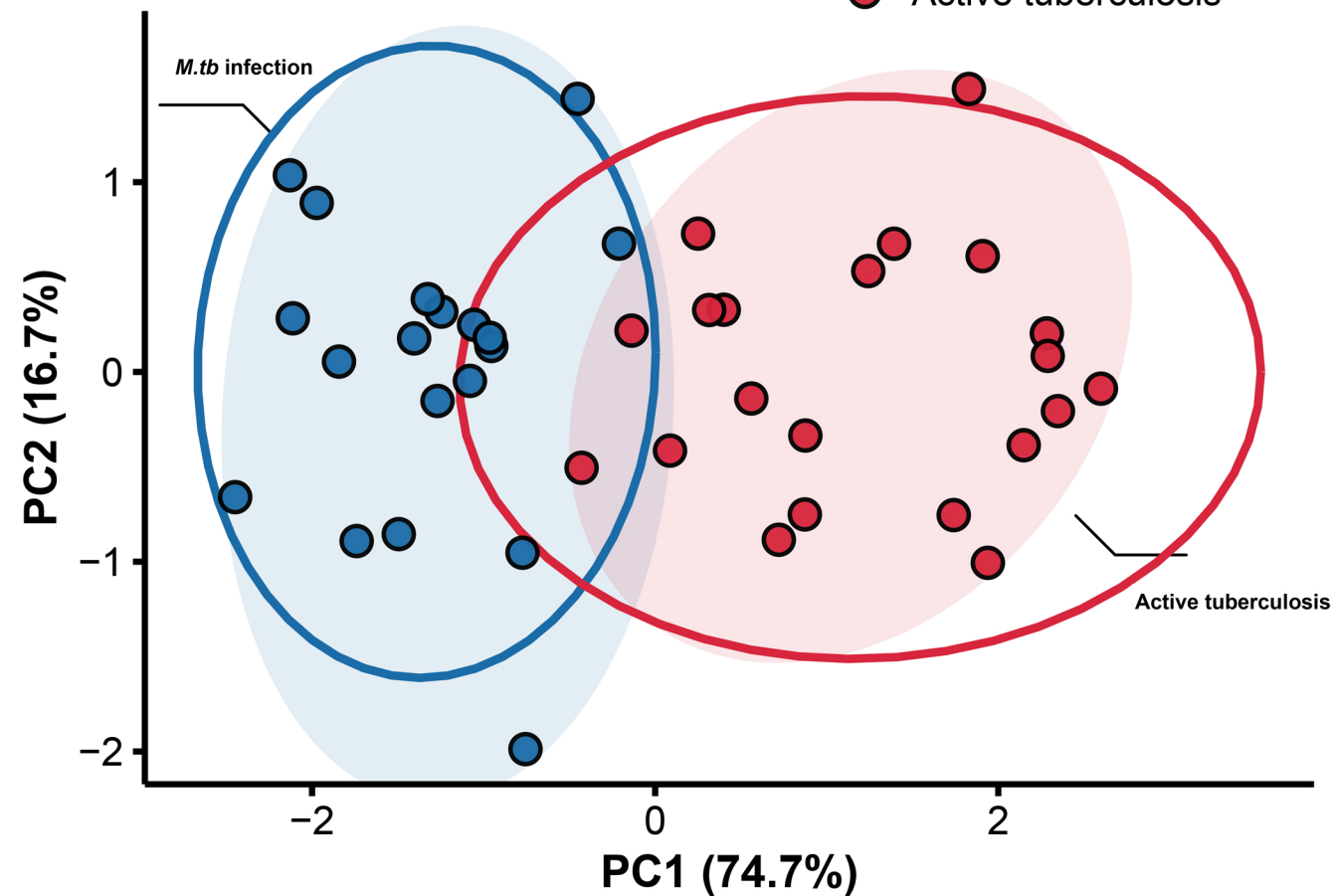

Supplement: SUPPLEMENTARY FIGURE 4 — Principal component analysis based on the prognostic proteomic signature demonstrates robust separation of individuals with latent infection and active tuberculosis. [file Data_Sheet_4.pdf]

# Decision Curve Analysis

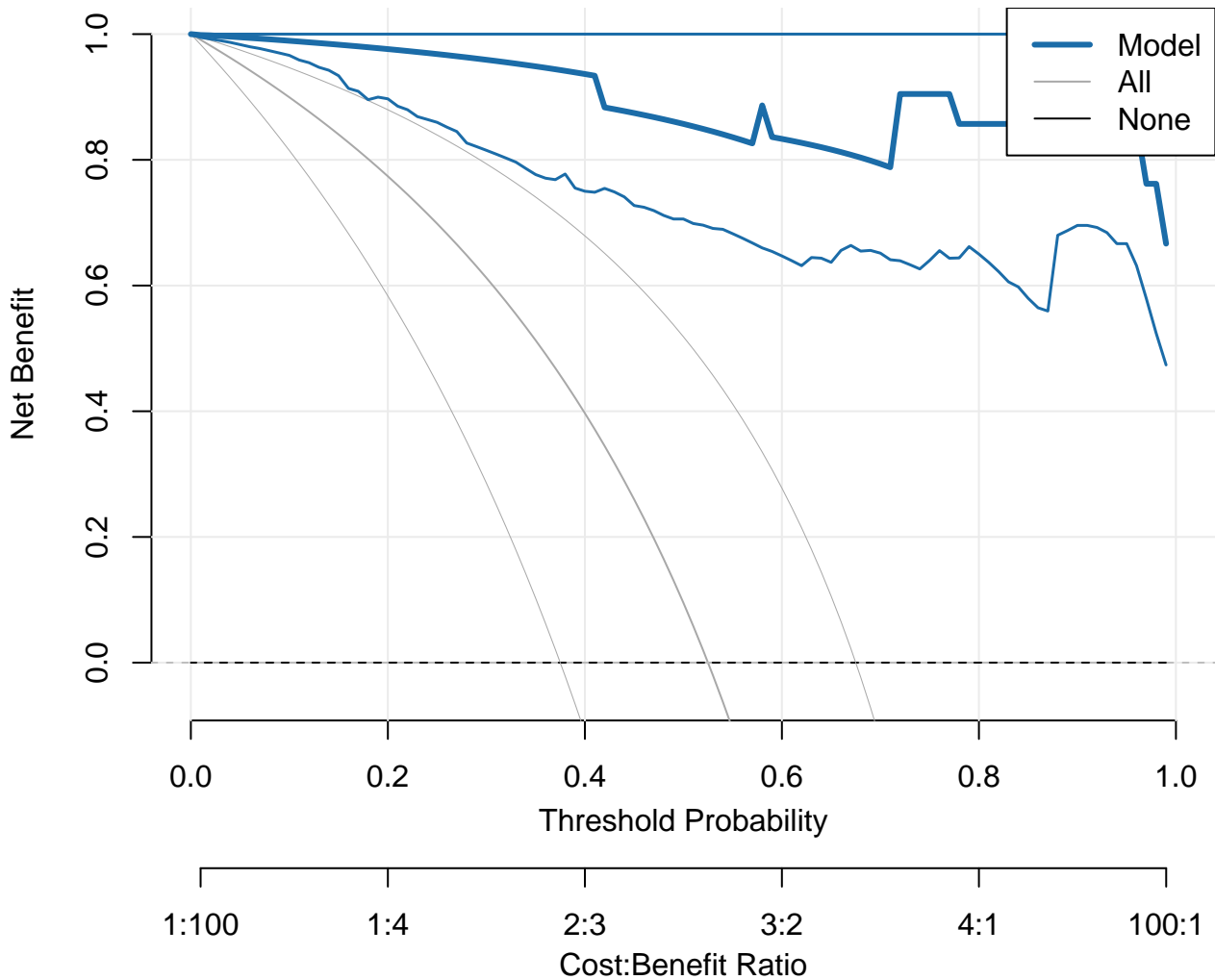

Supplement: SUPPLEMENTARY FIGURE 5 — Decision curve analysis confirms the clinical utility of the prognostic proteomic signature for guiding intervention decisions. [file Data_Sheet_5.pdf]
