# Supplementary material for: A plasma proteomic signature of the actin-coagulation axis accurately predicts progression to active tuberculosis
Source: Front Microbiol. 2026 Jan 26;16:1746190. doi: 10.3389/fmicb.2025.1746190 (PMC12883649; doi:10.3389/fmicb.2025.1746190)
Supplement: SUPPLEMENTARY FIGURE 2 — Preprocessing and normalization pipeline for the quantitative plasma proteomic data. (A) The right-skewed distribution of raw protein intensities. (B) The distribution after log₂ transformation. (C) The final approximately normal distribution after z-score normalization. [file Data_Sheet_2.pdf]

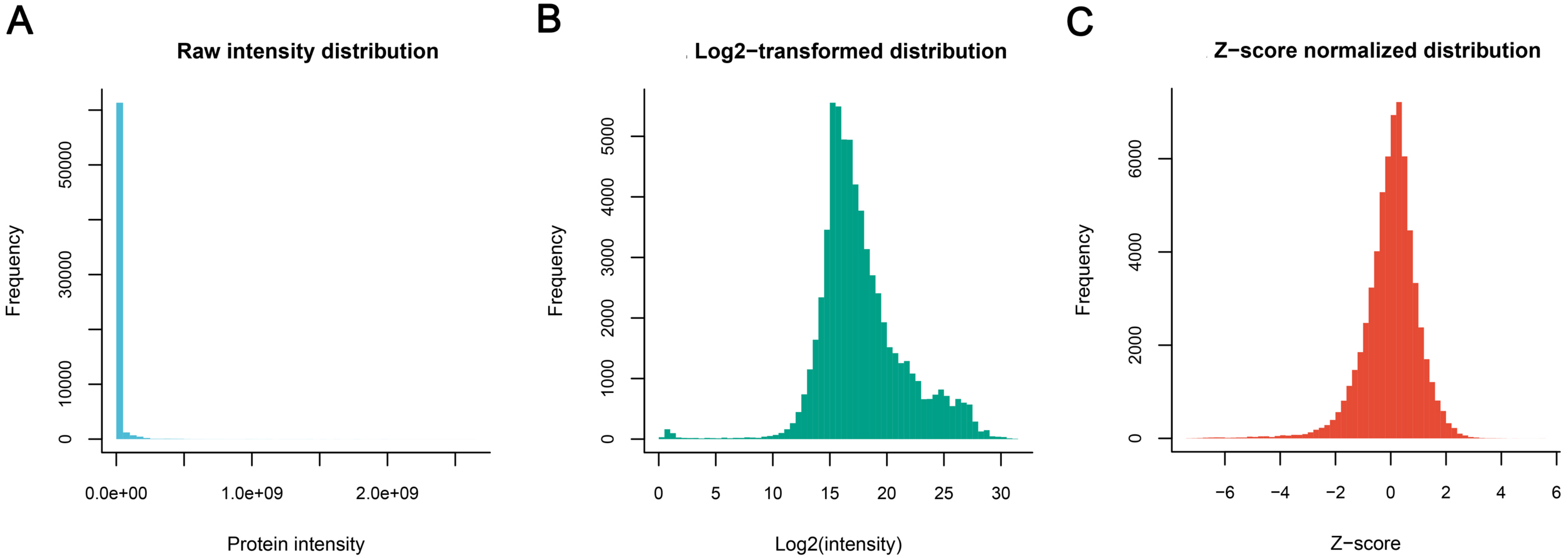
A
Raw intensity distribution
Frequency
0
10000
30000
50000
0.0e+00
1.0e+09
2.0e+09
Protein intensity
B
Log2−transformed distribution
Frequency
0
1000
2000
3000
4000
5000
0
5
10
15
20
25
30
Log2(intensity)
C
Z−score normalized distribution
Frequency
0
2000
4000
6000
−6
−4
−2
0
2
4
6
Z−score
