# Supplementary material for: A plasma proteomic signature of the actin-coagulation axis accurately predicts progression to active tuberculosis
Source: Front Microbiol. 2026 Jan 26;16:1746190. doi: 10.3389/fmicb.2025.1746190 (PMC12883649; doi:10.3389/fmicb.2025.1746190)
Supplement: SUPPLEMENTARY FIGURE 3 — Cross-validation performance of a random forest classifier for distinguishing among active tuberculosis, latent infection, and healthy states. (A) Confusion matrix showing classification accuracy for each class. (B) Top 10 most important protein features ranked by their contribution to the model. (C) Receiver operating characteristic (ROC) curves for each class, with corresponding area under the curve (AUC) values. (D) Boxplots of predicted probabilities for each true group. [file Data_Sheet_3.pdf]

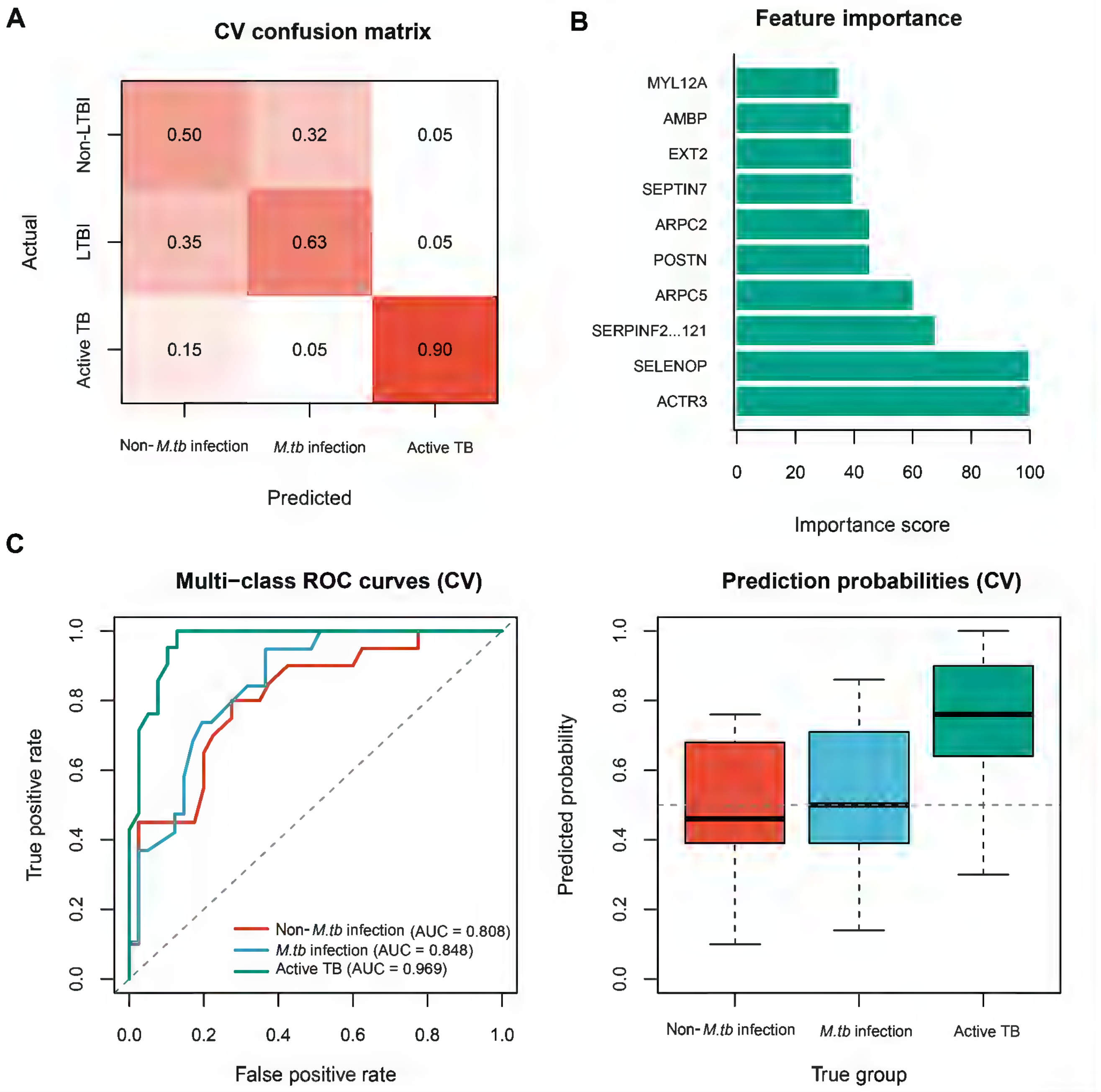

A
CV confusion matrix
Actual
Non-LTBI
LTBI
Active TB
0.50
0.32
0.05
0.35
0.63
0.05
0.15
0.05
0.90
Non-M.tb infection
M.tb infection
Active TB
Predicted
B
Feature importance
MYL12A
AMBP
EXT2
SEPTIN7
ARPC2
POSTN
ARPC5
SERPINF2...121
SELENOP
ACTR3
0
20
40
60
80
100
Importance score
C
Multi−class ROC curves (CV)
True positive rate
False positive rate
0.0
0.2
0.4
0.6
0.8
1.0
Non-M.tb infection (AUC = 0.808)
M.tb infection (AUC = 0.848)
Active TB (AUC = 0.969)
Prediction probabilities (CV)
Predicted probability
Non-M.tb infection
M.tb infection
Active TB
True group
